# A Higher Tidal Volume May Be Used for Athletes according to Measured FVC

**DOI:** 10.1155/2013/526138

**Published:** 2013-10-27

**Authors:** Pavlos Myrianthefs, George Baltopoulos

**Affiliations:** Faculty of Nursing, University of Athens, ICU at Agioi Anargyroi General Hospital, Nea Kifisia, 14561 Athens, Greece

## Abstract

We investigated whether professional athletes may require higher tidal volume (*T*_*v*_) during mechanical ventilation hypothesizing that they have significantly higher “normal” lung volumes compared to what was predicted and to nonathletes. Measured and predicted spirometric values were recorded in both athletes and nonathletes using a Spirovit SP-1 spirometer (Schiller, Switzerland). Normal *T*_*v*_ (6 mL/kg of predicted body weight) was calculated as a percentage of measured and predicted forced vital capacity (FVC) and the difference (*δ*) was used to calculate the additional *T*_*v*_ required using the equation: New *T*_*v*_(*T*_*v*_ *N*) = *T*_*v*_ + (*T*_*v*_ × *δ*). Professional athletes had significantly higher FVC compared to what was predicted (by 9% in females and 10% in males) and to nonathletes. They may also require a *T*_*v*_ of 6.6 mL/kg for males and 6.5 mL/kg for females during mechanical ventilation. Nonathletes may require a *T*_*v*_ of 5.8 ± 0.1 mL/kg and 6.3 ± 0.1 mL/kg for males and females, respectively. Our findings show that athletes may require additional *T*_*v*_ of 10% (0.6/6 mL/kg) for males and 8.3% (0.5/6 mL/kg) for females during general anesthesia and critical care which needs to be further investigated and tested.

## 1. Introduction: Background

Lung protective ventilation during the course of acute respiratory distress syndrome (ARDS) uses a tidal volume (*T*_*v*_) of 6 mL/kg of predicted body weight [1]. This *T*_*v*_ selection was associated with significant outcomes improvement in patients with ARDS [1]. 

This *T*_*v*_ was selected because normal lung volumes are predicted on the basis of sex and height [2, 3]. Also, this *T*_*v*_ corresponds to a normal seated man at rest which is 6 to 7 mL per kilogram [4]. Thus, it was attempted an association between *T*_*v*_ selection during mechanical ventilation and normal (predicted) lung volumes in healthy individuals at rest.

However, normal lung volumes may differ significantly between different ethnic populations and subpopulations who may have higher pulmonary function tests (PFTs) volumes including forced vital capacity (FVC) and forced expiratory volume in one second (FEV_1_) than average population independently from predicted body weight. Also, it is well known that PFTs are mainly based on sex, age, height, and weight. Finally, professional athletes have higher FVC and FEV_1_ than what was predicted for the same body weight and thus a higher *T*_*v*_ could be required to achieve “normal” *T*_*v*_.

None of the studies performed until now attempted to see the effect of different PFTs values and especially the FVC in this population on tidal volume selection. To answer this question we investigated the possible effect of measured versus predicted FVC values on tidal volume selection in professional athletes.

## 2. Subjects and Methods

For the study purposes an informed consent was taken by all participants for spirometry and the study protocol was approved by Hospital Advisory Board and local Ethics Committee Board.

### 2.1. Study Subjects: Participants

An urban population of Greece living in Athens (70 m above sea level) aged 20–65-year old were invited to participate in the study. These included students of Athens University, employees of our hospital and their families, and individuals visiting our outpatient clinic for checkup. Also, athletes were included from various sporting activities and spirometry was performed while visiting their facilities after communication with their trainer.

We explained the purpose of the study and the procedure of spirometry and then a clinical examination was performed based on a combination of the ECCS (European Community for Steel and Coal) standardized questionnaire on respiratory symptoms by the interviewing physician to identify eligible participants [5].

Exclusion criteria were unacceptable spirometry, previous or current smoking habit, history of chest injuries; chest, abdominal, oral, or facial pain, and presence of denture; exposure to substances known to cause lung injury; known respiratory disease (asthma, pulmonary tuberculosis, emphysema, or chronic bronchitis); respiratory symptoms during the last 12 months; hypertension; history of myocardial infarction; diabetes; dementia or confusional state; and the use of any drug and especially diuretics, cardiac glycosides, or *b*-adrenergic blocking agents [6].

Height was measured at the nearest 0.5 cm without shoes, in a standing position with the feet together, with the patient erect and looking straight ahead (Frankfort position). Subjects were weighted without shoes wearing indoor clothing. Age was also recorded according to birthday to the nearest 0.5 year. BMI and BSA were derived from height and weight.

### 2.2. Spirometry

Spirometry was performed following American Thoracic Society/European Respiratory Society (ATS/ERS) Task Force recommendations [5, 7, 8]. All tests were performed by two physicians well educated and experienced in spirometry. Spirometry and flow/volume loops were performed using a Schiller Spirovit SP-1 spirometer (Schiller, Switzerland). This spirometer is ATS/ERS approved, fulfilling the criteria for minimal recommendations for spirometry systems and calibrated regularly [7]. Spirometry was performed in sitting position in armed chairs wearing a nose clip. Subjects were relaxed and did not smoke, exercise, consume alcohol, wear heavy clothing, or eat large meal before testing. The procedure was performed at the same room between 8.00 and 10.00 am and barometric pressure, temperature, and relative humidity were registered every morning. Hygiene and infection control measures were undertaken as recommended [7].

At least three acceptable trials were required, defined as a good start of test (extrapolated volume of <5% of FVC or 0.15 L, whichever was larger), at least 6 s of expiration and a plateau in the volume/time curve (change in volume <30 mL for ≥2 s). As recommended by the ATS, data that did not meet reproducibility criteria were not excluded, but subjects were asked to perform up to a maximum of eight manoeuvres in an attempt to obtain reproducible results. The highest FEV_1_ and FVC from tests of acceptable quality were used for analysis [6].

### 2.3. Calculations

We had four groups of data that are males and females for athletes and nonathletes, respectively. Predicted body weight was calculated according to measured height for all participants using standard equations. For males it was calculated as equal to 50 + 0.91 (centimeters of height, 152.4), and that for females was calculated as equal to 45.5 + 0.91 (centimeters of height, 152.4) [1, 9]. Predicted *T*_*v*_(*T*_*v*_Pr = 6 mL/kg) was calculated according to predicted body weight [1]. Then, predicted *T*_*v*_ was then calculated as percentage of measured (Ms) and predicted (Pr) FVC values and their difference (*δ*, %) were extracted for each individual separately. This difference was used to calculate the new *T*_*v*_(*T*_*v*_ *N*) using the equation, New *T*_*v*_(*T*_*v*_ *N*) = *T*_*v*_ + (*T*_*v*_ × *δ*). New *T*_*v*_(*T*_*v*_ *N*) was divided to predicted body weight to calculate the corresponding *T*_*v*_ per kilogram separately for each individual.

### 2.4. Statistics

Values are expressed as mean ± SEM. A paired sample *t*-test was used for comparison of numerical data. A *P* value of <0.05 was used to define statistical significance.

## 3. Results

Of the 550 normal individuals (nonathletes) approached 235 met the inclusion criteria and were divided according to sex which resulted in two groups of 113 males and 122 females. Also, of the 315 professional athletes 251 met the inclusion criteria and were divided according to sex which resulted in two groups of 156 males and 95 females. Summary of the study population is shown in Table 1. Mean duration of sporting was 11.8 ± 6.4 and 11.6 ± 6.9 years for males and females, respectively. Male athletes were swimmers (*n* = 41), basketball players (*n* = 28), football players (*n* = 28), handball players (*n* = 13), athletics (*n* = 27), and gymnastic (*n* = 19). Female athletes were swimmers (*n* = 27), basketball players (*n* = 27), handball players (*n* = 25), athletics (*n* = 12), and gymnastic (*n* = 4).

Measured (Ms) and predicted (Pr) FEV_1_ and FVC were recorded and shown in Table 2. For nonathletes males measured FEV_1_ and FVC were significantly lower compared to what was predicted while for females measured FVC was significantly lower compared to what was predicted. For male and female athletes measured values were significantly higher from predicted values obtained from the ECSC prediction equation [10]. Also, in athletes measured values were approximately 9-10% higher than predicted PFTs values. The ratio (%) of measured/predicted values in athletes and nonathletes for FVC is shown in Table 3.

The predicted tidal volume according to the ARDS Network (*T*_*v*_Pr, 6 mL/kg), the percentage to measured (Ms) and predicted (Pr) FVC, their difference *δ*, and the new *T*_*v*_(*T*_*v*_ *N*) are shown in Table 4 separately for athletes, nonathletes, males, and females.

**Figure 1:**
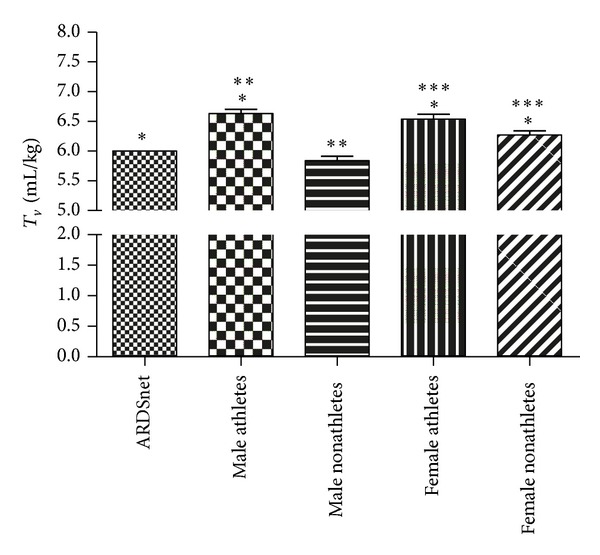
Comparison of ARDSnet tidal volume (6 mL/kg) to those according to athletic status and sex. *, **, and *** denote statistically significant differences.

Extracted *T*_*v*_ *N* according to FVCm was 6.6 ± 0.1 mL/kg (95% CI 6,5–6,8) for male athletes. For female athletes extracted *T*_*v*_ *N* was 6.5 ± 0.1 mL/kg (95% CI 6,4–6,7). Extracted *T*_*v*_ *N* was 5.8 ± 0.1 mL/kg (95% CI 5,7–6,0) for male nonathletes. For female nonathletes extracted *T*_*v*_ *N* was 6.3 ± 0.1 mL/kg (95% CI 6,1–6,4). See also Table 4 for details. Our calculated new tidal volumes (*T*_*v*_ *N*) were significantly higher compared to ARDS Network suggested tidal volumes (paired sample *t*-test, *P* < 0.0001) except for male nonathletes which was significantly lower (*P* ≤ 0.0156) Figure 1.

We observed that the additional tidal volume required for male athletes was 0.6 mL/kg which is 10% (0.6 mL/6 mL) of the suggested protective ventilation according to ARDS network. This 10% is in accordance with the 10.6% and 9.9% higher FVC and FEV_1_, respectively found in measured values compared to predicted values as shown in Table 2. Also, the additional tidal volume required for female athletes was 0.5 mL/kg which is 8.3% (0.5 mL/6 mL) of the suggested protective ventilation according to ARDS Network. This 8.3% is in accordance with the 8.9% and 7.8% higher FVC and FEV_1_, respectively, found in measured values compared to predicted values as shown in Table 2.

## 4. Discussion

In this study we found that athletes have significantly higher spirometric values compared to what was predicted and nonathletes. We also found that tidal volume during mechanical ventilation in athletes should be 6.6 mL/kg for males and 6.5 mL/kg for females compared to 6 mL/kg as suggested by the ARDS Network [1].

Early ventilation strategies in ARDS involved volume controlled ventilation with *T*_*v*_ of 10–15 mL/kg to achieve “normal” arterial blood gases [11]. However, ventilation itself can cause lung injury and after a landmark study by the ARDS Network a lung protective strategy using 6 mL/kg of ideal body weight was established leading to a 9% absolute mortality reduction along with reduced pulmonary and circulating inflammatory cytokines [1]. This study was confirmed by subsequent study in which patients that were ventilated with higher *T*_*v*_ and lower PEEP had increased ICU and hospital mortality [12].

A recent trial in patients with respiratory failure without ARDS also demonstrated low *T*_*v*_ ventilation to be protective, preventing ARDS, and associated with a reduction in the release of inflammatory cytokines. This study was stopped early due to an increased incidence of lung injury in patients ventilated with higher *T*_*v*_ [13].

Studies addressing several concerns regarding low *T*_*v*_ have shown that low *T*_*v*_ ventilation is a safe strategy and should be adopted in the management of patients with ARDS [14–16]. These studies demonstrate the importance of using lower *T*_*v*_ to ventilate the injured lung as opposed to aiming to normalize blood gases variables.

Low *T*_*v*_ (lung protective) strategy is a physiological approach using normal tidal volume which is at rest 6-7 mL/kg [4]. The ARDS network used this normal *T*_*v*_ (6 mL/kg) in relation to predicted body weight because normal lung volumes are predicted on the basis of sex and height in an attempt to synchronize *T*_*v*_ selection during MV in ARDS population and normal lung volumes [1].

In this study we compared predicted *T*_*v*_ according to ARDS Network calculations to measured and predicted FVC which are normal lung volumes. Our hypothesis was that since normal *T*_*v*_ is proportional to normal lung volumes, the comparison of normal *T*_*v*_ of 6 mL/kg as percentage to actual (measured) FVC could indicate the correct *T*_*v*_ appropriate for MV. According to our hypothesis normal *T*_*v*_ is a proportion of FVC and thus may be useful in the determination of “normal” *T*_*v*_ applied for MV instead of using predicted body weight.

We found also that measured spirometric volumes differ significantly from what was predicted according to athletic status. According to our hypothesis athletes having significantly higher FVC than what was predicted may require higher *T*_*v*_ than 6 mL/kg. We found that *T*_*v*_ may differ according to sex and athletics status.

We found that athletes may require an 8–10% increase in *T*_*v*_ that is 6.6 mL/kg for males and 6.5 mL/kg for females. This difference may result in a *T*_*v*_ of 462 mL instead of 420 mL for a 70 kg male athlete (42 mL higher). Also, for the same body weight of 70 Kg female athlete *T*_*v*_ would be 455 mL instead of 420 mL (35 mL higher).

On the other hand we found that according to measured FVC values nonathlete males may require slightly reduced *T*_*v*_ of 5.8 mL/kg and females of 6.3 mL/kg. This difference may result in a *T*_*v*_ of 406 mL instead of 420 mL for a 70 Kg male nonathlete (14 mL lower) and 441 mL instead of 420 mL for a female athlete (21 mL higher). Thus, it can be argued that for nonathletes the approach of 6 mL/kg is appropriate with a range of 5.8–6.3 mL/kg.

Comparing males there is a significant difference between athletes and nonathletes of 0.8 mL/kg (6.6–5.8). That is for a 70 kg male athlete a *T*_*v*_ of 462 mL is required and for a male nonathlete a *T*_*v*_ of 406 mL is required (*δ* = 56 mL). For females this difference is lower being 0.2 mL/kg (6.5–6.3) and of a female athlete the *T*_*v*_ would be 455 mL while for a female nonathlete the *T*_*v*_ would be 441 mL (*δ* = 14 mL).

In accordance with our study it was found larger lung capacity (FVC and FEV_1_) independent of age and height in never smokers with higher levels of physical exercise [17]. There also is evidence that even mild but also professional exercise is related to higher spirometric values and lower FEV_1_ loss over time [18–23]. These studies are in accordance with our findings that athletes have higher spirometric values to predicted [24].

It should be mentioned that currently in Europe, the reference equations for spirometry published by ECSC statement are used for people aged 18–70 yrs, with a height range of 155–195 cm in males and 145–180 cm in females [10]. The recent ATS/ERS Task Force committee does not recommend any specific set of equations for use in Europe but suggests the need for a new Europe-wide study to derive updated reference equations for lung function [8]. Also, suggests that the subjects being tested should be asked to identify their own race/ethnic group and even nation and recognises and encourages the continuing interest of worldwide researchers in deriving and using race/ethnic/nation-specific reference equations [8]. Finally, there are unexplained differences in lung function between ethnically similar nonsmoking symptom-free populations and centre variation between several European countries was found more likely to be due to true population differences [25]. This is in accordance with previous observations by us [26]. These issues have been recently addressed by the European Respiratory Society Global Lung Function Initiative research examining over 97,759 records of healthy nonsmokers aged 2.5–95 yrs [27].

Some limitations of our study should be underscored. Firstly, this is a hypothetical study based on our data that normal spirometric lung volumes differ among athletes and nonathletes and that athletes may require higher *T*_*v*_. There are no supporting data in the literature suggesting that the increase in *T*_*v*_ is related to the increase in FVC. Also, this was not a controlled clinical trial but an observational study. Most of our healthy adult participants were from mid to upper socioeconomic strata, so generalizing to other groups (especially to clinical populations such as patients with respiratory disease) is not advised. Also, we examined different sports and ages with different exercise duration and intensity. Finally *T*_*v*_ was measured during spirometry and thus calculated tidal volumes were not compared to what was calculated.

## 5. Conclusions

Professional athletes have significantly higher spirometric lung volumes compared to currently predicted values and those of nonathletes. According to measured FVC values of our population and our hypothesis, appropriate *T*_*v*_ may differ between athletes and nonathletes and could be 10% higher compared to ARDSnet recommendation. Additional *T*_*v*_ for professional athletes under mechanical ventilation and ARDS may be 0.6 mL/kg for males and 0.5 mL/kg for females. The tidal volume increase may be proportional to the percentage of FVC increase compared to predicted values. For nonathletes the ARDSnet recommendation maybe appropriate. Our findings need to be further investigated and tested.

## Figures and Tables

**Table 1:** Summary statistics for males and females.

Variable	Nonathletes	Athletes
Males	*n* = 113	*n* = 156

Age (years)	38.3 ± 12.9	26.1 ± 0.8
Weight (kg)	86.1 ± 13.2	79.4 ± 1.1
Height (cm)	177.7 ± 6.6	180.7 ± 0.7

Females	*n* = 122	*n* = 95

Age (years)	41.7 ± 13.9	24.1 ± 0.8
Weight (kg)	66.5 ± 10.5	64.1 ± 1.0
Height (cm)	161.4 ± 7.2	171.7 ± 0.8

**Table 2:** Measured and predicted spirometric values (mL).

Males	Measured	Predicted	*δ*
	Athletes, *n* = 156		
FVC	5808 ± 81.9*	5252 ± 49.5*	+555.9 ± 61.7
FEV_1_	4831 ± 58.9*	4396 ± 40.9*	+434.8 ± 45.8
FEV_1_/FVC	83.69 ± 0.48*	82.07 ± 0.12*	+1.6 ± 0.47
	Nonathletes, *n* = 113		
FVC	4715 ± 70.0**	4856 ± 53.9**	−141.2 ± 55.3
FEV_1_	3876 ± 58.4**	4006 ± 48.5**	−129.9 ± 45.2
FEV_1_/FVC	82.8 ± 0.6*	80.6 ± 0.2*	+2.2 ± 0.6

Females	Measured	Predicted	*δ*

	Athletes, *n* = 95		
FVC	4364 ± 70.8*	4008 ± 46.3*	+355.9 ± 55.0
FEV_1_	3757 ± 57.3*	3484 ± 40.2*	+273.4 ± 45.8
FEV_1_/FVC	86.34 ± 0.52*	83.73 ± 0.09*	+2.6 ± 0.50
	Nonathletes, *n* = 122		
FVC	3294 ± 62.1*	3157 ± 51.8*	+137.0 ± 35.6
FEV_1_	2779 ± 54.8	2717 ± 48.2	+61.9 ± 32.9
FEV_1_/FVC	84.3 ± 0.6*	81.6 ± 0.2*	+2.7 ± 0.6

FVC: forced vital capacity, FEV_1_: forced expiratory volume in one second, m: measured, and pr: predicted according to ECSC equation. [10] *P < 0.0001, **P < 0.05.

**Table 3:** Ratio (%) of measured/predicted values in athletes and nonathletes for FVC.

	FVCm/FVCpr
Male athletes	110.6 ± 1.1%
Male nonathletes	97.4 ± 1.1%
Female athletes	109.0 ± 1.4%
Female nonathletes	104.5 ± 1.1%

FVC: forced vital capacity, m: measured, and pr: predicted.

**Table 4:** Calculations to extract new *T*_*v*_(*T*_*v*_ *N*) according to measured (Ms) and predicted (Pr) PFTs.

Males	Predicted (mL)	% FVCms	% FVCpr	*δ* (%)	*T*_*v*_ *N* (mL)	*T*_*v*_ *N* (mL/Kg)
Athletes, *n* = 156						
*T*_*v*_	454.4 ± 3.97	7.9 ± 0.86*	8.6 ± 0.03*	0.7 ± 0.08	502.7 ± 6.9	6.6 ± 0.07

Nonathletes, *n* = 113						
*T*_*v*_	438.3 ± 3.4	9.5 ± 0.12*	9.1 ± 0.06*	−0.3 ± 0.01	426.0 ± 5.5	5.8 ± 0.07

Females	Predicted (mL)	% FVCm	% FVCpr	*δ* (%)	*T*_*v*_ *N* (mL)	*T*_*v*_ *N* (mL/Kg)

Athletes, *n* = 95						
*T*_*v*_	375.4 ± 4.6	8.7 ± 0.12*	9.4 ± 0.05*	0.6 ± 0.01	408.9 ± 7.1	6.5 ± 0.08

Nonathletes, *n* = 122						
*T*_*v*_	319.3 ± 3.6	9.9 ± 0.15*	10.3 ± 0.09*	0.3 ± 0.01	333.1 ± 4.9	6.3 ± 0.07

FVC: forced vital capacity, Ms: measured, and Pr: predicted. *P < 0.0001, **P < 0.001.

## Abbreviations

PFTs: Pulmonary Function Tests
FVC: Forced Vital Capacity
FEV_1_: Forced Expiratory Volume in One Second
ALI/ARDS: Acute Lung Injury/Acute Respiratory Distress Syndrome
ATS/ERS: American Thoracic Society/European Respiratory Society
BMI: Body Mass Index
BSA: Body Surface Area
ECCS: European Community for Steel and Coal.
